# APBioNet—Transforming Bioinformatics in the Asia-Pacific Region

**DOI:** 10.1371/journal.pcbi.1003317

**Published:** 2013-10-31

**Authors:** Asif M. Khan, Tin Wee Tan, Christian Schönbach, Shoba Ranganathan

**Affiliations:** 1 Perdana University Graduate School of Medicine, Selangor Darul Ehsan, Malaysia; 2 Department of Pharmacology and Molecular Sciences, The Johns Hopkins University School of Medicine, Baltimore, Maryland, United States of America; 3 Department of Biochemistry, Yong Loo Lin School of Medicine, National University of Singapore, Singapore; 4 School of Science and Technology, Department of Biology and Chemistry, Nazarbayev University, Astana, Republic of Kazakhstan; 5 Department of Bioscience and Bioinformatics and Biomedical Informatics R&D Center (BMIRC), Kyushu Institute of Technology, Fukuoka, Japan; 6 Department of Chemistry and Biomolecular Sciences and ARC Centre of Excellence in Bioinformatics, Macquarie University, Sydney, Australia

## Introduction

The Asia-Pacific Bioinformatics Network (APBioNet; www.apbionet.org) is a nonprofit, nongovernmental, international organization founded in 1998 that focuses on the promotion of bioinformatics in the Asia-Pacific region. APBioNet's mission, since its inception, has been to pioneer the growth and development of bioinformatics awareness, training, education, infrastructure, resources, and research among member countries and economies. Its work includes technical coordination, liaison, and/or affiliation with other international scientific bodies, such as the European Molecular Biology network (EMBnet) and the International Society for Computational Biology (ISCB). APBioNet has more than 20 organizational and 2,000 individual members from over 12 countries in the region, from industry, academia, research, government, investors, and international organizations. APBioNet is spearheading a number of key bioinformatics initiatives in collaboration with international organizations, such as the Asia-Pacific Advanced Network (APAN), the Association of South-East Asian Nations (ASEAN), the Asia-Pacific Economic Cooperation (APEC), and the Asia-Pacific International Molecular Biology Network (A-IMBN), and industry partners. Many of the initiatives and activities have been initiated through its flagship conference, the International Conference on Bioinformatics (InCoB). In 2012, APBioNet was incorporated in Singapore as a public limited liability company to ensure quality, sustainability, and continuity of its mission to advance bioinformatics across the region and beyond. We describe below the key thrust areas of APBioNet.

## InCoB—Connecting the Bioinformatics Community in the Region

InCoB is a conference series that started in Bangkok, Thailand in 2002. Since then, APBioNet has adopted InCoB as its annual signature event (2003: Penang, 2004: Auckland, 2005: Busan, 2006: New Delhi, 2007: HongKong/Hanoi, 2008: Taipei, 2009: Singapore, 2010: Tokyo, 2011: Kuala Lumpur, and 2012: Bangkok) and grown it to become one of Asia's largest bioinformatics conferences, targeting practitioners from both biology and computing backgrounds (http://bit.ly/10P6CVq). In 2011, InCoB celebrated its tenth anniversary in Kuala Lumpur, Malaysia jointly with the 1^st^ ISCB-Asia [1] and featured several key initiatives, including the launch of the BioDB100 initiative, aimed at gathering 100 bioinformatics databases that are Minimum Information About a Bioinformatics investigation (MIABi)-standards compliant under one interoperable framework (http://incob.apbionet.org/incob11). InCoB2012 (http://incob.apbionet.org/incob12) marked the homecoming of the conference to its origin, Bangkok, as a reputable and major annual bioinformatics event in the Asia-Pacific region. The 2013 conference was held recently for the first time in China (http://incob.apbionet.org/incob13), with the 2014 conference to be held immediately prior to and sharing keynote speakers with the International Union for Pure and Applied Biophysics [IUPAB] 2014 Congress (http://incob.apbionet.org/incob14; July 31 to August 2, 2014), in Sydney, Australia.

## Advancing Standards for Bioinformatics Activities

In a multi-stakeholder effort to advance standards for bioinformatics activities, APBioNet has been working since InCoB2009 on building 100 exemplar biological and bioinformatics databases (BioDB100) and software tools (BioSW100) [2] that test the following aspects of standardization: i. data and software persistence and the basis for perpetuity (http://docid.apbionet.org); ii. reinstantiability and reproducibility (http://biodb100.apbionet.org); iii. author and contributor identity disambiguation (http://aid.apbionet.org); and iv. Minimum Information About a Bioinformatics investigation (MIABi) to include basic information necessary for an *in silico* experiment to be repeatable and the results reproducible, as well as harmonized with community initiatives in MIBBI [3] and those of the International Society for Biocuration (ISB; http://biocurator.org), specifically BioDBcore (http://biocurator.org/biodbcore.shtml). We anticipate that the ongoing testing efforts [4]–[7] will help to delineate the process for standardization and enhance the implementation of increasingly standardized vocabularies, ontologies, and infrastructural and informational interoperability for the maintenance and sustainability of knowledge resources of ever-increasing sophistication.

## Bioinformatics Education and Training

APBioNet has been actively engaged in bridging the bioinformatics capability gap, with emphasis on establishing sustainable bioinformatics education and training [8]. APBioNet was represented at the 2001 Workshop on Education in Bioinformatics (WEB), a satellite meeting of the International Conference on Intelligent Systems for Molecular Biology (ISMB) that provided, for the first time, a platform for bioinformatics educators to discuss fundamental educational and pedagogical issues for bioinformatics degree and training programs. APBioNet played a key role in the third East Asia Bioinformation Network meeting (http://eabn.apbionet.org) held in Singapore (2008) that witnessed the proposal for minimum skills required of biologists in bioinformatics and biocomputation (msrBIC) [8]. APBioNet cooperates with organizations, such as the S* Life Science Informatics Alliance (www.apbionet.org/s-star) [9] and the ASEAN Virtual Institute of Science and Technology (AVIST) (www.avist.org), to facilitate online/distance bioinformatics education and training. As a result of a research grant from the International Development and Research Centre (IDRC) of Canada and subsequent R&D at the National University of Singapore (NUS), APBioNet produced a bioinformatics grid-enabled software, as well as a distributable LiveOS containing bioinformatics software to facilitate bioinformatics training (http://en.wikipedia.org/wiki/BioSLAX). As part of its outreach, since 2003 APBioNet has partnered with various organizations to run bioinformatics workshops in the region (http://bit.ly/12eWyDe). In June 2012, APBioNet participated the inaugural Bioinformatics, Biotechnology, Biocuration and Computational Biology networks and societies (B^3^CB) meeting in Sweden that led to the establishment of the Global Organization for Bioinformatics Learning, Education and Training (GOBLET; http://mygoblet.org) to coordinate bioinformatics training activities worldwide. All these efforts have contributed to the generation of skilled bioinformaticians in the region.

## Database/Computational Services and Resources

One of the earliest projects that APBioNet set up was a collaboration to build a bioinformatics network riding on the advanced network infrastructure of the Asia-Pacific Advanced Network (APAN) (www.apbionet.org/APAN/apan-apbionetMar98.html). This led to the formation of the BioMirrors project (www.bio-mirror.net) [10] and a peer-to-peer (P2P) database replication system for low-bandwidth institutions [11]. In response to an increasing demand for high-performance and high-throughput computational biology, in 2001 APBioNet partnered with the NUS Bioinformatics Centre to promote the concept of grid computing in life sciences at the BioGrid'01 symposium (www.bic.nus.edu.sg/biogrid/biogrid01). More recently, APBioNet has been actively engaged in tracking the progress of advanced networking, such as the TransEurasia Information Network (TEIN2), developing cutting-edge initiatives, such as the International Workshop on World Wide Workflow Grid (GridAsia 2007; www.euasiagrid.org), and exploring applications of grid and cloud computing for life scientists (EUAsiaGrid BioWorkshop 2010; http://trg.apbionet.org/euasiagrid). APBioNet is committed to continuing these efforts to meet the challenges of big data science in the decade ahead [5].

## Policy and Awareness

Constructive scientific activism raises awareness among policy makers for the need to catalyze the transformation of life science research and education in the Asia-Pacific. APBioNet has worked closely with the ASEAN Committee on Science and Technology (COST) to assist in developing bioinformatics masterplans and roadmaps for the ten ASEAN member countries. Through the ASEAN Dialogue Partner mechanism, APBioNet is also actively engaging China, India, Japan, and Korea. Beyond these, APBioNet has also provided assistance to institutions in Pakistan and Saudi Arabia. As a result of these extensive, collective, and cooperative efforts to influence policy and raise awareness among policy makers and scientific leaders, Asian countries such as Singapore [12], Malaysia [13], Thailand [14], India (http://dbtindia.nic.in/annual05-06/Ch-8-eng.pdf), the Philippines, Korea, Pakistan, Indonesia, Brunei, and many others [15] have had strong growth of bioinformatics and its allied disciplines over the last decade [16], [17].

## Conclusions

APBioNet has come a long way since its inception in 1998 at the Pacific Symposium for Bioinformatics, Hawaii. Currently, it is the largest regional bioinformatics organization in the Asia-Pacific, and one of the oldest. It continues to expand its presence in the region by actively reaching out to the research and education community, through its flagship conference InCoB. APBioNet is also taking steps toward setting standards and influencing scientific policy to enable a new generation of scientists to embrace the new biology of today that is increasingly information- and technology-driven, with knowledge generation dependent on applications of physical and computer sciences.
